# Jenner

**Published:** 1896-07

**Authors:** 


### JENNER.

In considering the history of the discovery of vaccination, one is struck by the special preparation through which Jenner had passed, peculiarly fitting him for his life-work. For this discovery was not a matter of accident, a moment's insight and the thing completed, the preventive properties of the cow-pox having long been known to the milkmaids of his district without benefit to others. It required something more than this barren knowledge: it required Jenner's training, grafted upon his exceptional character, to stop the greatest scourge of his time. His love of natural history could not have received a stronger impetus nor his studies a better oversight than in the house of John Hunter, with its menagerie and specimens of all descriptions. His intercourse with such a man, which continued until Hunter's death, must have exerted great and beneficial influence on the pupil's mind. "Do not think, but try; be patient, be accurate," was some of the advice which he received; and Jenner thought and tried for years before he was satisfied with his investigations, which culminated one hundred years ago, on May 14th, in the first vaccination. Before this time smallpox destroyed, maimed, or disfigured about one-fourth of mankind. In England, the average annual mortality from smallpox per million, during the ten years between 1885 and 1894, was 26, while last century it was fully 2,000, and the decrease has been similar in other countries. That this is not due to a "natural decline of smallpox" is evident from its virulence in countries where the people are not properly vaccinated, such as in Russia and in Spain. While in 1889 the rate in Germany was 4, that in Almeria was 3,080.

The recent outbreak among the anti-vaccinators of Gloucester, England, Jenner's own county, speaks for itself.

Anti-vaccinators have urged that improved sanitation accounts for the admitted low mortality of smallpox in these days, but no unprejudiced person can accept that theory. Prussia and Austria had advanced in about equal degrees in sanitary matters, but in 1874 vaccination became compulsory in Prussia, with a sudden and striking fall in the death-rate from smallpox. Austria has shown no such change in the rate of mortality. Further, improved sanitation affects all ages alike, while the reduction in smallpox mortality has not affected all ages alike. Before primary vaccination, the burden of smallpox fell chiefly upon infancy; but vaccination has thrown that back to a greater age, and now more adults die of smallpox than in the early days of vaccination. What is necessary for a practically complete immunity throughout life is a second vaccination during school-life. In a well vaccinated community, such as that by which the Prussian army (in which revaccination is compulsory) has been surrounded since 1875, revaccinated masses of people have been proved to be practically absolutely safe against smallpox. When the present mistaken ideas of freedom which prevail in certain countries have given way to an understanding that every man is to some extent his brother's keeper, and that infectious disease affecting him is a menace to others, we shall have not only vaccination, but revaccination compulsory. It lies with the medical profession to see that the above facts are recognized by the public, and to urge the adoption of a vaccination law.

Jenner opened the door for all the later inoculators. While they are striving to prevent and cure the host of the remaining ailments of the human and lower races, it is pitiable to see his, the greatest discovery of all, still, at the end of a hundred years, bearing only a little of the fruit it ought to bear, and that from a mixture of ignorance and prejudice among the laity, and a lack of energy among medical men, who take care, however, that they themselves are freely vaccinated.

To all who feel an interest in Jenner and his work, we recommend the *British Medical Journal* for May 23d, which contains half a dozen good pictures of the famous physician himself, and others of the place of his birth, his memorial window, etc., as well as *fac simile* reproductions of letters of interest. Over fifty pages are given up in this "centenary number" to Jenner and vaccination, and among its most valuable contributions are the thirty chromographic reproductions of George Kirkland's colored drawings of vaccinia and inoculated variola, placed side by side at the various stages of their development. These represent conditions nearly precisely similar up to the third day. On the fourth, the already slightly greater reaction on the smallpox arm is becoming more marked. This becomes daily visibly accentuated, till on the 8th the large yellow patch is surmounted by numerous new vesicles and much inflammation, both of which develop for six days longer, when the drying-up stage begins, and by the sixteenth day inflammation has nearly gone and hard crusts alone remain.

---

DR. SAMUEL LLOYD, of New York, who will be pleasantly remembered by those who were present at the recent meeting of the State Association in Augusta, on account of his social as well as of his professional qualities, has contributed a full review of that meeting to the New York *Postgraduate*. He concludes by saying: "Looking back at the meeting one cannot but feel that the average of the work done was of a most superior character. It compares favorably with any meeting of the New York State Medical Society that I have attended, and there can be no question that, aside from the rest that one gets by a trip to these State Societies, a great deal of information may be gathered if the rest of the State Societies are of an equally high average as that of the State of Georgia."

---

IT is reported that a lady has presented the French Academy with 800,000 francs, the interest of which is to go to any one who will discover a cure for consumption. It says a good deal for the high reputation for disinterestedness of physicians among the laity that there has not long ago dangled such a prize before the medical eye for every known dangerous or painful disease. While quite agreeing that doctors are the least mercenary of mankind, we should yet welcome international subscription lists with such an object, for the desire for a life-long competence might stimulate to research some who otherwise would merely jog along. Still more profitable would it be if such funds were invested in the support of more numerous pathological laboratories and of the men who work in them.

